# Atypical Bacilliredoxin AbxC Plays a Role in Responding to Oxidative Stress in Radiation-Resistant Bacterium *Deinococcus radiodurans*

**DOI:** 10.3390/antiox10071148

**Published:** 2021-07-20

**Authors:** Soyoung Jeong, Jong-Hyun Jung, Min-Kyu Kim, Arjan de Groot, Laurence Blanchard, Sangryeol Ryu, Yong-Sun Bahn, Sangyong Lim

**Affiliations:** 1 Radiation Research Division, Korea Atomic Energy Research Institute, Jeongeup 56212, Korea; soyoung@kaeri.re.kr (S.J.); jungjh83@kaeri.re.kr (J.-H.J.); mkkim@kaeri.re.kr (M.-K.K.); 2 Department of Food and Animal Biotechnology, Research Institute of Agriculture and Life Sciences, Seoul National University, Seoul 08826, Korea; sangryu@snu.ac.kr; 3 Department of Radiation Science and Technology, University of Science and Technology, Daejeon 34113, Korea; 4 Molecular and Environmental Microbiology Team, Aix Marseille Univ, CEA, CNRS, BIAM, 13108 Saint Paul-Lez-Durance, France; nicolaas.degroot@cea.fr (A.d.G.); laurence.blanchard@cea.fr (L.B.); 5 Department of Biotechnology, College of Life Science and Biotechnology, Yonsei University, Seoul 03722, Korea

**Keywords:** *Deinococcus radiodurans*, oxidative stress, bacilliredoxin, bacillithiol, DR_1832

## Abstract

*Deinococcus radiodurans* is a robust bacterium with extraordinary resistance to ionizing radiation and reactive oxygen species (ROS). *D. radiodurans* produces an antioxidant thiol compound called bacillithiol (BSH), but BSH-related enzymes have not been investigated. The *D. radiodurans* mutant lacking *bshA* (*dr*_*1555*), the first gene of the BSH biosynthetic pathway, was devoid of BSH and sensitive to hydrogen peroxide (H_2_O_2_) compared to the wild-type *D*. *radiodurans* strain. Three bacilliredoxin (Brx) proteins, BrxA, B, and C, have been identified in BSH-producing bacteria, such as *Bacillus*. *D*. *radiodurans* possesses DR_1832, a putative homolog of BrxC. However, because DR_1832 contains a novel signature motif (TCHKT) and a C-terminal region similar to the colicin-like immunity domain, we named it AbxC (atypical BrxC). The deletion of *abxC* also sensitized cells to H_2_O_2_. AbxC exhibited peroxidase activity in vitro, which was linked to nicotinamide adenine dinucleotide phosphate (NADPH) oxidation via the BSH disulfide reductase DR_2623 (DrBdr). AbxC proteins were present mainly as dimers after exposure to H_2_O_2_ in vitro, and the oxidized dimers were resolved to monomers by the reaction coupled with BSH as an electron donor, in which DrBdr transported reducing equivalents from NADPH to AbxC through BSH recycling. We identified 25 *D. radiodurans* proteins that potentially interact with AbxC using AbxC-affinity chromatography. Most of them are associated with cellular metabolisms, such as glycolysis and amino acid biosynthesis, and stress response. Interestingly, AbxC could bind to the proposed peroxide-sensing transcription regulator, DrOxyR. These results suggest that AbxC may be involved in the H_2_O_2_ signaling mechanism mediated by DrOxyR.

## 1. Introduction

Reactive oxygen species (ROS) are harmful byproducts of aerobic metabolism. ROS include superoxide anions (O_2_^•−^), hydrogen peroxide (H_2_O_2_), and hydroxyl radicals (^•^OH), which have inherent chemical reactivity [1]. Oxidative stress caused by excess ROS generation leads to damage to cellular components, including lipids, proteins, and DNA [2]. To cope with the adverse effects of ROS, bacteria have evolved elaborate antioxidant systems [2]. These include ROS scavenging enzymes, such as catalase (CAT), superoxide dismutase (SOD), and alkyl hydroperoxide reductase (AhpCF), and redox-active enzymes such as thioredoxin (Trx) and glutaredoxin (Grx) [3,4]. Trx acts as an efficient thiol–disulfide reductant, which provides reducing equivalents to peroxiredoxin (Prx), which in turn acts as a peroxidase capable of catalyzing H_2_O_2_ [5].

*Deinococcus radiodurans* is well known for its extreme resistance to ionizing radiation (IR). Since IR exposure leads to ROS production, resulting in damage to cellular macromolecules, *D. radiodurans* is also resistant to oxidative stress [6]. *D. radiodurans* encodes three SODs (DR_1279, DR_1546, and DR_A0202), two CATs (DR_1998 and DR_A0259) and four Prxs (DR_0846, DR_1208, DR_1209, and DR_2242), among which DR_1279 (named SodA) and DR_1998 (named KatE1) are constitutively present at high levels [7,8]. Alkyl hydroperoxide reductase C (AhpC) belongs to the typical 2-Cys Prx with two highly conserved cysteine residues: N-terminal peroxidatic CysP and C-terminal resolving CysR [9]. In *D. radiodurans*, AhpE (DR_2242), classified as atypical 1-Cys Prx containing only the CysP (lost the CysR), was detected with the AhpD-like protein (DR_1765), which protects cells from oxidative stress as a substitute for AhpF [10,11]. *D. radiodurans* possesses two Trxs (DR_0944 and DR_A0164) with the classic active site motif CGPC. DR_A0164 is reduced by *D. radiodurans* Trx reductase (TrxR; DR_1982), which can utilize only NADPH for activity [12]. DR_0846 is a Trx-dependent PrxQ, a homolog of bacterioferritin comigratory protein (BCP), which contains the two closely situated Cys residues (CXXXXC), and it functions as a molecular chaperone as well as a peroxidase [13].

**Figure 1:**
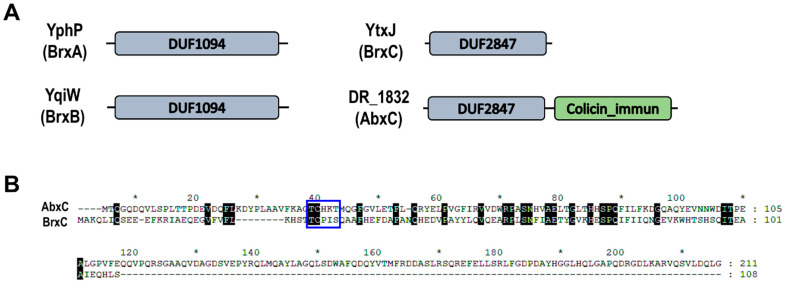
Comparison of bacilliredoxin (Brx) proteins from *Bacillus subtilis* and DR_1832 from *Deinococcus radiodurans*. (**A**) Schematic comparison of the domain structures of Brxs and atypical BrxC (AbxC). The C-terminal extended region (colicin_immun) of DR_1832 (atypical BrxC; AbxC) has similarity with bacterial self-protective colicin-like immunity domain (PF09204). (**B**) Comparison of amino acid sequences of AbxC and *B. subtilis* BrxC. Alignment was performed using Clustal Omega, and protein sequences were retrieved from the Uniprot database (www.uniprot.org. Accessed 8 June 2021): Q9RTD3_DEIRA (DR_1832) and YtxJ_BACSU (BrxC). White letters on black shading represent 100% identity. The blue line box indicates the TCHKT and TCPIS motif conserved in AbxC and BrxC, respectively.

Most aerobic organisms generate low-molecular-weight thiols (LMW thiols) such as glutathione (GSH), mycothiol (MSH), and bacillithiol (BSH), which are involved in the maintenance of cellular redox potentials and the protection of cells from a variety of reactive chemical and electrophilic species [14]. The best studied LMW thiol is the tripeptide GSH present in all eukaryotes and Gram-negative bacteria [15]. However, some Gram-positive Firmicutes such as *Bacillus* and *Staphylococcus* species and *D. radiodurans* lack GSH and instead produce BSH, an α-anomeric glycoside of l-cysteinyl-d-glucosamine with l-malic acid, which is functionally analogous to GSH [16]. Under oxidizing conditions, BSH forms BSH disulfide (BSSB), and the flavine adenine dinucleotide (FAD) cofactor-containing BSSB reductase Bdr (previously named YpdA) reverts it from its oxidized form using NADPH [17,18]. BSH also forms mixed disulfides with protein thiols, termed *S*-bacillithiolation, as a means of modulating or protecting protein activity against over-oxidation, and its reversal is catalyzed by bacilliredoxin (Brx) proteins [19]. In *Bacillus subtilis*, the redox sensitive organic hydroperoxide repressor (OhrR) is bacillithiolated in response to both cumene hydroperoxide (CHP) and sodium hypochlorite (NaOCl) treatment, suggesting a function for BSH in redox sensing [15,19]. Three Brx proteins have been identified in BSH-producing bacteria: YphP (renamed BrxA), YqiW (BrxB), and YtxJ (BrxC) (Figure 1A). BrxA and BrxB are paralogs of the DUF1094 family with an unusual CXC redox motif, which is different from the classical redox active CXXC motif found in Trx or Grx. The function of BrxA and BrxB in protein de-bacillithiolation has been demonstrated for the *S*-bacillithiolated OhrR and two metabolic enzymes, methionine synthase MetE and glyceraldehyde-3-phosphate dehydrogenase Gap, in *B*. *subtilis* or *S*. *aureus* [20,21]. Both BrxA and BrxB likely use a monothiol mechanism to reduce *S*-bacillithiolated proteins, in which the N-terminal Cys thiolate of the Brx CGC motif attacks the *S*-bacillithiolated protein, resulting in the reduction of the mixed disulfide and formation of an *S*-bacillithiolated Brx (Brx-SSB). Brx-SSB is then reduced by BSH, leading to BSSB formation, which is subsequently reduced by Bdr with electrons from NADPH [17]. BrxC belonging to the Trx-like protein DUF2847 family is a monothiol Brx with the active site Cys located in a conserved TCPIS motif reminiscent of that found in monothiol Grx [12,22]. Recently, the *B. subtilis* BrxC protein has been shown to catalyze the de-bacillithiolation of BrxB and Bdr in vitro and GapDH in vivo [23]. 

*D. radiodurans* encodes a putative homolog of Bdr (DR_2623), which shows 39% and 35% identities with *B. subtilis* and *S. aureus* Bdr proteins, respectively [10]. The FAD-binding domain of Bdr contains the canonical glycine-rich signature sequence motif GXGXXG [24], which is conserved in DR_2623 (Figure S1). Regarding Brx, *D. radiodurans* possesses only the BrxC-type protein DR_1832 [12]. The DUF2847 domains of DR_1832 and *B. subtilis* BrxC share 25.24% identity. However, DR_1832 is longer than BrxC because of the presence of a C-terminal region that has similarity with the bacterial self-protective colicin-like immunity domain (PF09204) (Figure 1A), and the TCPIS motif observed in BrxC is replaced with TCHKT in DR_1832 (Figure 1B). The basic local alignment search tool for proteins (BLASTP) revealed that all deinococcal DR_1832 homologs have the C-terminal extension (data not shown), and the signature motif TCHKT is strictly conserved in all of these homologs (Figure S2). Therefore, DR_1832 was named AbxC (atypical BrxC). In this study, we investigated AbxC biochemically to establish a functional AbxC/BSH/DR_2623/NADPH redox cycle and found that AbxC exerts BSH-dependent peroxidase activity and interacts with the proposed *D. radiodurans* peroxide responsive regulator DrOxyR.

## 2. Materials and Methods

### 2.1. Culture Conditions

*Deinococcus radiodurans* R1 strain (ATCC13939) and its isogenic mutant strains were cultivated at 30 °C in TGY broth (0.5% tryptone, 0.1% glucose, and 0.3% yeast extract) with aeration or on TGY plates supplemented with 1.5% Bacto-agar. *Escherichia coli* strain DH10B was used as the host for gene cloning. *E. coli* strains were grown at 37 °C in Luria–Bertani (LB) medium (Difco Laboratories, Detroit, MI, USA) or on LB plates solidified with 1.5% Bacto-agar. Antibiotics were added to the medium if necessary: ampicillin, 100 μg/mL (*E. coli*), kanamycin, 5 μg/mL (*D. radiodurans*), and chloramphenicol, 3.8 μg/mL (*D. radiodurans*).

### 2.2. Construction of Mutants and Plasmids

The *bshA* (*dr*_*1555*) and *abxC* (*dr*_*1832*) mutant strains were constructed by targeted mutagenesis using the double crossover recombination method described previously [25,26]. Upstream and downstream regions (approximately 1 kb) of the coding sequences of target genes were amplified by polymerase chain reaction (PCR) using sets of primers carrying restriction sites for cloning (Table S1). Each PCR fragment was digested with the appropriate restriction enzymes (Table S1) and cloned into the corresponding sites of plasmid pKatAPH3 [27]. For transformation with the resulting plasmid, *D. radiodurans* cells from exponentially growing cultures were collected and concentrated 50-fold in TGY supplemented with 30 mM calcium chloride (CaCl_2_). Cell mixture containing the constructed plasmid DNAs was held on ice and then incubated at 32 °C for 90 min. The positive mutants were selected on TGY agar plates supplemented with kanamycin as described previously [28]. The disruption of the target genes was confirmed by diagnostic PCR and subsequent DNA sequencing.

The *abxC* gene expression plasmid pAbxC_WT_ was constructed using the pRADZ3 shuttle vector, which contains the *groEL* promoter for constitutive gene expression and functions both in *E*. *coli* and *D*. *radiodurans* [8]. This plasmid is present in the cell at approximately the same copy number as the chromosome, which is present at 7 to 10 copies per cell [29]. The complete *abxC* coding sequence was PCR-amplified from the genomic DNA of *D. radiodurans* R1 using 1832-F and 1832-R primers, which carry the ApaI and EcoRV restriction sites, respectively (Table S1). The substitution of cysteine to serine at position 36 (C36S) was introduced into AbxC using the complementary primer pair C36S-F/R containing the C36S mutation (Table S1). Site-directed mutagenesis was performed directly on pAbxC_WT_ using QuikChange II Site-Directed Mutagenesis Kit^TM^ (Agilent Technologies, La Jolla, CA, USA). The amplified plasmids (named pAbxC_C36S_) were digested with DpnI and transformed into *E. coli* DH10B. The C36S substitution was verified by sequencing. The transformed *D. radiodurans* cells were selected using chloramphenicol (3.8 μg/mL) supplementation.

### 2.3. Measurement of BSH

BSHs were measured by high-performance liquid chromatography (HPLC) analysis of fluorescent thiol adducts with monochlorobimane (mBCl), as described previously [30]. Cells grown to log phase (OD_600_ ≈ 1.0) were harvested and resuspended in extraction buffer (50% acetonitrile, 2 mM mBCl and 20 mM Tris-HCl pH 8.0). After incubation at 60 °C for 15 min, acidification with 25 mM methanesulfonic acid was followed by centrifugation to remove the aggregated proteins. Thiol–bimane conjugates from the supernatants were subjected to HPLC analysis with a fluorescence detector (λex = 385 nm, λem = 460 nm) installed in the Agilent 1200 HPLC system (Agilent technologies). ).

### 2.4. Survival Assay

A stationary-phase culture that had grown overnight was inoculated into fresh TGY broth at a 1:100 dilution. Cells grown to log phase (OD_600_ ≈ 1.0) in TGY broth without antibiotics were adjusted to OD_600_ ≈ 0.1 with TGY and then challenged with 20, 40, and 60 mM H_2_O_2_ for 1 h at 30°C. Following treatment with catalase (Sigma-Aldrich, Saint Louis, CA, USA) to remove residual H_2_O_2_, the cells were serially diluted 10-fold in distilled water and spotted onto solid TGY medium. The TGY plates were incubated at 30 °C for 2–3 days to enumerate the colony forming units (CFU).

### 2.5. Protein Carbonylation Assay

Cells grown to log phase (OD_600_ ≈ 1.0) were treated with 0 or 40 mM H_2_O_2_ for 1 h and harvested by centrifugation. The cell suspension in phosphate buffered saline (PBS) was lysed by sonication, and the total protein concentration in the supernatants was measured using the Bradford assay. Carbonyl group quantification was performed using the traditional 2,4-dinitrophenylhydrazine (DNPH) method using the Protein Carbonyl Colorimetric Assay Kit (Cayman Chemical, Ann Arbor, MI, USA). Derivatization of protein carbonyls by DNPH was followed by absorbance measurements at 370 nm, according to the manufacturer’s instructions.

### 2.6. Protein Purification

Coding sequences of *abxC*, *drBdr* (*dr*_*2623*), *drOxyR* (*dr*_*0615*), *dr*_*1022*, and *dr*_*1262* genes were PCR-amplified using the primer pair for each gene (Table S1) and cloned into the pET-21a vector harboring a His × 6 tag at the C-terminus (Novagen, Darmstadt, Germany). In addition, the *abxC* gene PCR product was ligated to the plasmid pET28_FLAG_ containing a 3×FLAG-tag sequence at the N-terminus [31] to produce FLAG-tagged AbxC protein. *E. coli* BL21-CodonPlus (DE3)-RP strain (Novagen) was transformed with the resulting plasmids and cultivated in LB broth (0.5 L) with shaking (200 rpm) at 37 °C until the culture density reached 0.5-0.6 of OD_600_. Protein expression was induced with 0.2 mM Isopropyl β-d-1-thiogalactopyranoside (IPTG), and the cultures were further incubated at 16 °C for 20 h. Collected cells were suspended in 20 mL of buffer A (20 mM Tris-HCl pH 7.5, 200 mM NaCl) and disrupted by pressuring twice at 40 kpsi using the OS Cell Disrupter (Constant Systems Ltd., Northants, UK). After centrifugation, 15 mL of crude extract was applied to 5 mL of Ni-NTA resin (Qiagen, Valencia, CA, USA). The resin was washed with 50 mL of buffer A containing 30 mM imidazole and eluted with buffer A containing 300 mM imidazole. The samples were concentrated using an Amicon Ultra filter unit (30,000 NMWL, Merck Millipore, Burlington, MA, USA), loaded on a Superdex 75HR column, and eluted with buffer A in an AKTA FPLC system (GE Healthcare Life Science, Boston, MA, USA). The fractions containing the target proteins were verified using sodium dodecyl sulfate–polyacrylamide gel electrophoresis (SDS-PAGE) and concentrated using an Amicon Ultra filter unit (Merck Millipore). Protein concentration was determined using the Bradford method with bovine serum albumin as a standard.

### 2.7. NADPH Consumption Assay

The electron transfer activity of DrBdr with BSH and AbxC was determined by measuring NADPH consumption in 50 mM Tris HCl (pH 8.0) at room temperature (RT). Reaction mixtures consisting of 500 μM NADPH, 500 μM BSH (Carbosynth LLC, San Diego, CA, USA), and 0.5 μM DrBdr were treated with 10 mM H_2_O_2_ in the absence or presence of 10 μM of AbxC. NADPH consumption was monitored immediately after the start of the reaction as absorbance change at 340 nm using a microplate reader (TECAN, Mannedorf, Switzerland). The reaction was also initiated by adding oxidized AbxC (for a final concentration of 10 μM), which was prepared by incubation with a 10-fold molar excess of thiol-oxidizing agent diamide in 50 mM Tris-HCl (pH 8.0) for 60 min [32].

### 2.8. Quantification of Thiols

Free sulfhydryl (-SH) groups of AbxC were quantitatively measured using the 5,5-dithio-bis-2-nitrobenzoic acid (DTNB) reduction assay. DTNB is reduced by free thiols in an exchange reaction, resulting in the formation of mixed disulfide between analytes and TNB release; hence, it has been used as a classical chromogenic reagent for thiol detection [33]. Purified AbxC protein was added to 50 mM Tris-HCl buffer (pH 7.5) containing 2 mM ethylene-diamine-tetraacetic acid (EDTA) to obtain a final concentration of 0.1 µM and then treated with different concentrations of H_2_O_2_. The reaction was initiated by adding DTNB (at a final concentration of 10 mM) at RT, and the increase in absorbance at 412 nm by 2-nitro-5-thiobnzoic acid (TNB) release was monitored using a microplate reader (TECAN) until fluorescence plateaus were reached. The free -SH concentration was determined by comparison with a standard curve composed of known concentrations of cysteine.

### 2.9. Western Blotting

To express FLAG-tagged AbxC protein in *D. radiodurans*, the plasmid pKatAPH3 harboring the upstream and downstream regions of *abxC* (pKatAPH3-UD1832) used for the *abxC* mutant (Δ*abxC*) construction was modified. To introduce the FLAG tag sequence in front of the *abxC* ATG codon, in the first step, two fragments were amplified using the primer pairs 1832-Up-F/1832-Flag-R and 1832-Flag-F/1832-R (Table S1) from genomic DNA and pET28_FLAG_ containing *abxC*, respectively. In the second step, the two PCR products were annealed at their overlapping homologous regions and amplified using the 1832-Up-F/1832-R primer pair. The fusion PCR product was cloned into pKatAPH3-UD1832. A kanamycin-resistance gene cassette of the resulting plasmid was swapped with a chloramphenicol-resistance gene cassette from pKatCAT5 [34] by EcoRV and BamHI digestion and subsequent ligation. The plasmid with the sequential array of the upstream region of *abxC*, *abxC* with a FLAG epitope on the N-terminus, chloramphenicol resistance gene, and downstream region of *abxC* was introduced into Δ*abxC*.

For Western blot analysis, *D. radiodurans* cells expressing FLAG-tagged AbxC were grown in TGY medium to OD_600_ ≈ 1.0, harvested by centrifugation, and then resuspended in PBS. Following sonication for cell lysis, the total protein concentration in the supernatants was measured using the Bradford assay. Total protein (5 µg) was resolved by SDS-PAGE under non-reducing conditions and subsequently transferred to a polyvinylidene fluoride (PVDF) membrane in a Mini Trans-Blot Cell (Bio-Rad, Hercules, CA, USA) at 100 V for 2 h in Novex Bolt^TM^ transfer buffer (Thermo Scientific, Waltham, MA, USA). After blocking in 5% skim milk in Tris-buffered saline with Tween 20 (TBS-T) for 1 h at RT, the PVDF membrane was incubated with mouse anti-FLAG antibody (1:5000; cat. no. F2555; Sigma-Aldrich; Merck KGaA) for 1h at RT and sequentially probed with horseradish peroxide (HRP)-conjugated rabbit anti-mouse IgG antibody (1:5000; cat. no. A9044; Sigma-Aldrich). The secondary antibody was detected using a tetramethylbenzidine (TMB) substrate reagent according to the manufacturer’s instructions (BD Biosciences, San Jose, CA, USA). The chemiluminescent signals on the PVDF membrane were visualized using a ChemiDoc image system (Bio-Rad). Far-Western blotting was performed as described above, except that 5 µg of each purified protein DrOxyR, DR_1022, and DR_1262 was loaded on a 12% SDS-PAGE gel and incubated with FLAG-tagged AbxC (0.21 nM) at 25°C for 2 h after transfer. Following washing with TBS-T three times, the PVDF membrane was incubated with mouse anti-FLAG antibody (1:5000) for 1h at RT and sequentially probed with HRP-conjugated rabbit anti-mouse IgG antibody (1:5000). The secondary antibody was detected using the TMB substrate reagent (BD Biosciences) [35].

### 2.10. Preparation of AbxC Affinity Column and Capturing of Target Proteins

AbxC was immobilized on a resin and used to capture potential target proteins in cellular extracts. The affinity column of AbxC was prepared according to the procedure of Sturm et al. (2009) [36]. *D*. *radiodurans* cell lysate (10 mg) from an exponential phase culture was incubated with AbxC-bound beads overnight at 4 °C. Beads were washed with 100 mM Tris (pH 8.0) containing 0.5 M NaCl and then eluted using 10 mM dithiothreitol (DTT). Proteins in the eluate were separated using 12% SDS-PAGE. The bands of interest were excised from the gel and digested in-gel with trypsin. The eluted peptides were analyzed by nanoscale liquid chromatography–tandem mass spectrometry (nLC-MS-MS) using a dual-cell linear ion trap Orbitrap mass spectrometer (LTQ Velos; Thermo Scientific) installed at the National Center for Interuniversity Research Facilities (NCIRF) at Seoul National University (Seoul, Korea). Proteins were identified by searching the MS/MS spectra against a protein database of *D. radiodurans* using SEQUEST (version 27, Thermo Scientific) [37,38].

## 3. Results and Discussions

### 3.1. A BSH-Deficient D. radiodurans Strain Is Sensitive to H_2_O_2_

**Figure 2:**
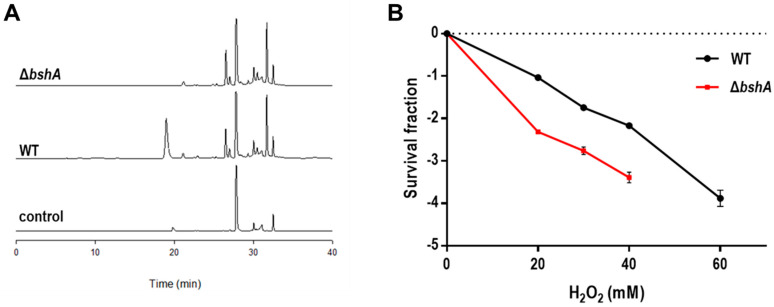
Characterization of *D. radiodurans bshA* mutant strain (Δ*bshA*). (**A**) Representative HPLC analysis of BSH in the *D. radiodurans* wild-type strain (WT) and Δ*bshA*. BSH was derivatized with monochlorobimane (mBCl) and analyzed by HPLC. Cell extraction buffer was used as control. The HPLC analysis of cellular thiols was repeated two times, with no significant difference in the results. (**B**) Survival of Δ*bshA* exposed to H_2_O_2_. WT and Δ*bshA* were treated with H_2_O_2_ at the indicated concentrations for 1 h. The survival fraction was calculated by dividing the colony forming units (CFU) of H_2_O_2_ treated cells by the CFU of non-treated cells. Values are means and standard deviations of two independent experiments conducted in duplicate.

In *D. radiodurans*, three Bsh enzymes BshA (DR_1555), BshB (DR_2363a; WP_010888991.1), and BshC (DR_1647) are known to be responsible for BSH biosynthesis [10]. We deleted the *bshA* gene in *D. radiodurans* to construct a BSH-deficient strain (Δ*bshA*). Δ*bshA* did not show any alteration in growth in comparison to the wild-type *D. radiodurans* strain (WT) under normal conditions (Figure S3). When cells were labeled with monochlorobimane (mBCl), BSH formed a complex with mBCl to generate the monobromobimane derivative of BSH [16]. The BSH–bimane derivative peak was observed at 19 min in the WT cell extracts but disappeared in the HPLC chromatogram of Δ*bshA* (Figure 2A), indicating that BSH production was abolished in Δ*bshA*. The protective role of BSH in response to oxidative stress was estimated by measuring the survival rate of Δ*bshA* following H_2_O_2_ treatment. Δ*bshA* was more sensitive to H_2_O_2_ than WT; Δ*bshA* showed an approximately 1-log reduction in cell survival compared to that of WT in the presence of 40 mM H_2_O_2_ (Figure 2B), suggesting that BSH is involved in the antioxidant systems of *D. radiodurans*. This result is consistent with the observation that *S. aureus bshA* mutants are sensitized to exogenous H_2_O_2_ [39].

### 3.2. Mutation of abxC Decreases H_2_O_2_ Resistance of D. radiodurans

**Figure 3:**
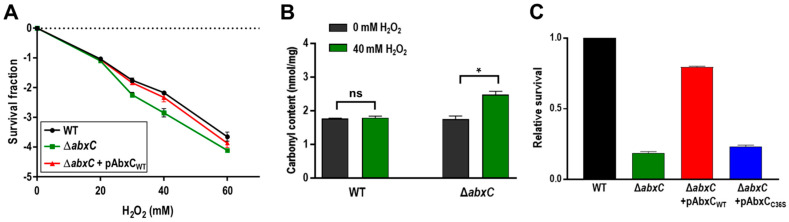
Effects of *abxC* mutation on H_2_O_2_ resistance. (**A**) Survival of Δ*abxC* exposed to H_2_O_2_. WT, Δ*abxC*, and Δ*abxC* harboring pAbxC_WT_ that encodes the wild-type AbxC were treated with H_2_O_2_ at the indicated concentrations for 1 h. The survival fraction was calculated by dividing the CFU of H_2_O_2_-treated cells by the CFU of non-treated cells. Values are means and standard deviations of two independent experiments conducted in duplicate. (**B**) Protein carbonylation of Δ*abxC* in response to H_2_O_2_. After treatment of 40 mM H_2_O_2_ for 1 h, the carbonylated protein contents of WT and Δ*abxC* were measured by the 2,4-dinitrophenylhydrazine (DNPH) assay. Data represent the means ± standard deviations of three independent experiments. Comparisons between two groups were performed by Student’s t-test (GraphPad Prism 7.04). A *p*-value of less than 0.01 is considered statically significant (ns; not significant, *; *p* < 0.01). (**C**) Complementation of Δ*abxC*. WT, Δ*abxC*, and Δ*abxC* harboring the pAbxC_WT_ and pAbxC_C36S_ plasmids, which produce the wild-type AbxC and its C36S mutant proteins, respectively, were treated with 40 mM H_2_O_2_ for 1 h. Survival abilities of Δ*abxC* mutants were expressed relative to WT survival (arbitrarily set at 1), i.e., the CFU of mutants were normalized to the CFU of WT. Data represent the means ± standard deviations of three independent experiments.

To investigate the role of AbxC in response to H_2_O_2_, we constructed a *abxC* mutant strain (Δ*abxC*) and examined its survival rates under oxidative stress conditions. The viability of ∆*abxC* cells was lower than that of the WT following H_2_O_2_ treatment (Figure 3A). The carbonyl content was increased in ∆*abxC* relative to that in WT by 40-mM H_2_O_2_ treatment (Figure 3B). We constructed an expression plasmid producing AbxC and used this construct to complement the Δ*abxC* strain. Complementation by plasmid-borne *abxC* in trans restored the WT level of growth in Δ*abxC* (Figure 3C). AbxC contained only one Cys residue in the putative catalytic motif (Figure 1B). The Cys residue (Cys36) of AbxC was substituted with serine using site-directed mutagenesis. When the engineered protein harboring C36S was provided in trans, it failed to restore the survival ability of Δ*abxC* exposed to H_2_O_2_ to WT levels (Figure 3C). These results suggest that the Trx-like protein AbxC contributes to *D. radiodurans* resistance to oxidative stress, and Cys36 plays a crucial role in the anti-oxidative function of AbxC.

### 3.3. AbxC Exhibits H_2_O_2_ Scavenging Activity

**Figure 4:**
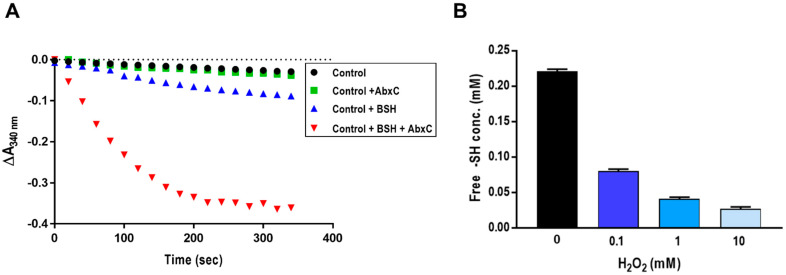
AbxC oxidation by scavenging H_2_O_2_. (**A**) NADPH consumption by the DrBdr/BSH/AbxC pathway. The control reaction mixture contained 500 µM NADPH and 0.5 µM DrBdr (DR_2623), and 500 µM BSH and/or 10 μM AbxC were added in the control mixture. The NADPH consumption was monitored at 340 nm immediately after addition of 10 mM H_2_O_2_. The experiments were repeated twice and representative data are shown. (**B**) Evaluation of free thiols (-SH) of AbxC. The purified AbxC (0.1 µM) treated with 0.1, 1, and 10 mM H_2_O_2_ was incubated with 10 mM DTNB solution. The TNB^2−^ ions released during these reactions were monitored by visible spectroscopy at 412 nm. The –SH concentrations were quantified by the use of a cysteine standard curve. Data represent the means ± standard deviations of three independent experiments.

BSH reacts directly with ROS, leading to the oxidation of BSH to BSSB [18]. *Bacillus* and *Staphylococcus* Bdr proteins have been recently shown to function as NADPH-dependent BSSB reductase [17,23,24]. To examine the BSSB reductase activity of DR_2623, a putative Bdr homolog, we analyzed the catalytic activity of purified DR_2623 (here designated DrBdr) in a BSH/NADPH-coupled assay in the presence of H_2_O_2_. The purified DrBdr had the characteristic yellow color of FAD-containing enzymes. Only a small amount of NADPH was consumed in the presence of BSH (Figure 4A). Interestingly, however, the addition of AbxC resulted in significant and fast consumption of NADPH as measured by a rapid absorbance decrease at 340 nm (Figure 4A). Cys36, residing in the conserved TCHKT motif, is the sole cysteine of AbxC (Figure 1B). The DTNB assay for thiol detection showed that AbxC lost its free thiols upon H_2_O_2_ treatment (Figure 4B), suggesting that AbxC could catalyze H_2_O_2_ reduction. Although DrBdr seemed to have no effect on BSSB reduction in the absence of AbxC, this could be attributed to the fact that the oxidation of BSH by ROS is strongly influenced by the reduction potential of oxidants [18]. In contrast to the strong oxidant hypochlorous acid (HOCl), which oxidizes BSH to mostly BSSB (70%), H_2_O_2_ oxidizes only about 10% of BSH to exclusively BSSB under the given experimental conditions [18]. In our assays, AbxC significantly enhanced NADPH consumption by DrBdr in response to oxidative stress induced by H_2_O_2_, and the NADPH consumption did not take place when either DrBdr or BSH was absent in the assay (Figure 4A and Figure S4). These results suggest that DrBdr has BSSB reductase activity and acts in the AbxC/BSH/DrBdr redox cycle for BSH regeneration.

### 3.4. H_2_O_2_ Stimulates AbxC Dimerization

**Figure 5:**
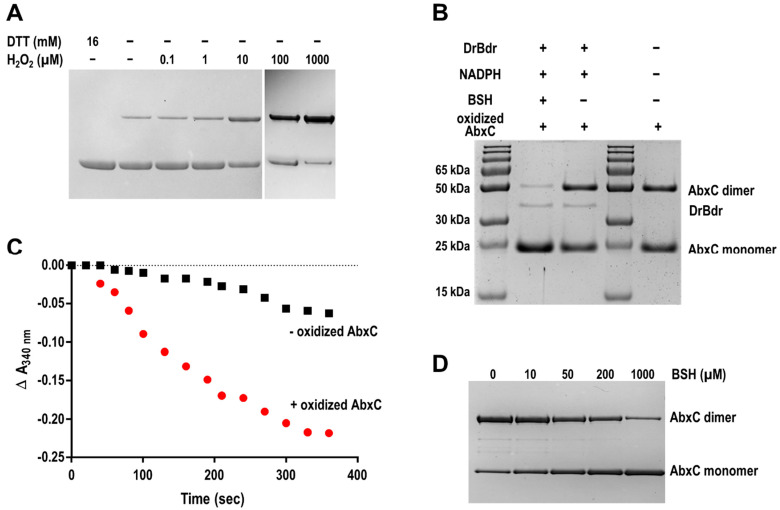
Dimer–monomer transition property of AbxC. (**A**) Effect of H_2_O_2_ on AbxC oxidation. The purified AbxC proteins (0.5 mg/mL) were incubated with increasing concentrations of H_2_O_2_ in PBS for 15 min and then separated using non-reducing SDS-PAGE (12% gels). DTT was used for AbxC reduction. (**B**) Effect of BSH in reduction of oxidized AbxC. AbxC proteins (0.5 mg/mL) oxidized by diamide treatment were incubated alone or with DrBdr (0.5 µM)/NADPH (500 µM)/BSH (500 µM) for 15 min in 20 mM Tris-HCl pH 7.5 at room temperature. The samples were analyzed by non-reducing SDS/PAGE. Bands representing the AbxC monomers and dimers and DrBdr are indicated. (**C**) NADPH consumption by oxidized AbxC. The reaction mixtures contained 500 µM BSH, 500 µM NADPH, and 0.5 µM DrBdr. Time-dependent consumption of NADPH was monitored at 340 nm immediately after addition of AbxC (− oxidized AbxC) or oxidized AbxC (+ oxidized AbxC). The experiments were repeated twice and representative data are shown. (**D**) Effect of BSH on AbxC reduction. AbxC proteins (0.5 mg/mL) were oxidized by diamide treatment and incubated with the indicated concentrations of BSH for 15 min in 20 mM Tris-HCl containing 500 µM NADPH. The mixtures were separated using non-reducing SDS-PAGE (12% gels).

To reveal the state of Cys in AbxC under oxidative stress conditions, AbxC was treated with H_2_O_2_, and the products were analyzed by non-reducing SDS-PAGE. Two bands for AbxC were observed in the absence of H_2_O_2_ (Figure 5A). The molecular weights of the upper and lower bands were equivalent to those of the AbxC monomer (23.7 kDa) and its homo-dimer (47.4 kDa), respectively (Figure 5B). The density of the upper band was increased, and that of the lower band was reduced in an H_2_O_2_-dependent manner (Figure 5A). This indicated that H_2_O_2_ oxidized the AbxC thiols (Cys36) to unstable Cys sulfenic acid intermediates (Cys-SOH), leading to AbxC dimerization through the formation of intermolecular disulfide bonds. This was supported by the disappearance of the upper band by the addition of a disulfide reducing agent dithiothreitol (DTT) (Figure 5A). The oxidized AbxC dimers prepared by incubation with excessive diamide were subjected to the analytical method of non-reducing SDS-PAGE gel. The electron transfer reaction including BSH, DrBdr, and NADPH resulted in a decrease in the density of the upper band, with a concomitant increase in the density of the lower band (Figure 5B). In addition, NADPH consumption was observed during the reaction (Figure 5C). These results demonstrate that recycling oxidized AbxC requires the BSH/DrBdr/NADPH redox pathway.

Under oxidizing conditions, the *Corynebacterium glutamicum* MSH peroxidase (Mpx) is recycled by the mycoredoxin (Mrx)/MSH/MSH disulfide reductase (Mtr) and/or Trx/TrxR electron pathways [40]. The glutathione peroxidase (Gpx) is recycled by GSH. The reduction of oxidized Gpx begins with a reaction with GSH to form GS-Gpx, and the removal of GSH from Gpx occurs either directly via a thiol–disulfide exchange reaction with another GSH molecule or is catalyzed by Grx [41]. The reduction mechanism of AbxC seems different from that of thiol-based antioxidant enzymes. AbxC was oxidized by H_2_O_2_ to form disulfide-bonded dimers, which could be reduced directly by BSH in conjunction with DrBdr (Figure 4 and Figure 5). BSH ionization is one of the unusual biophysical properties of BSH. BSH has a lower thiol pKa value than Cys or GSH, such that the availability of the reactive thiolate form (BS^−^) of BSH required for thiol–disulfide exchange reactions is not limited at physiological pH [42]. Thus, it is possible that BSH acts as a nucleophilic thiol available for reaction with disulfides such as DTT. BSH was able to convert the AbxC dimer to its monomeric form in a concentration-dependent manner even in the absence of DrBdr (Figure 5D), which confirmed that oxidized AbxC is non-enzymatically reduced by BSH. Further research is needed to delineate the mechanism underlying AbxC reduction.

### 3.5. Identification of Proteins Potentially Interacting with AbxC

**Figure 6:**
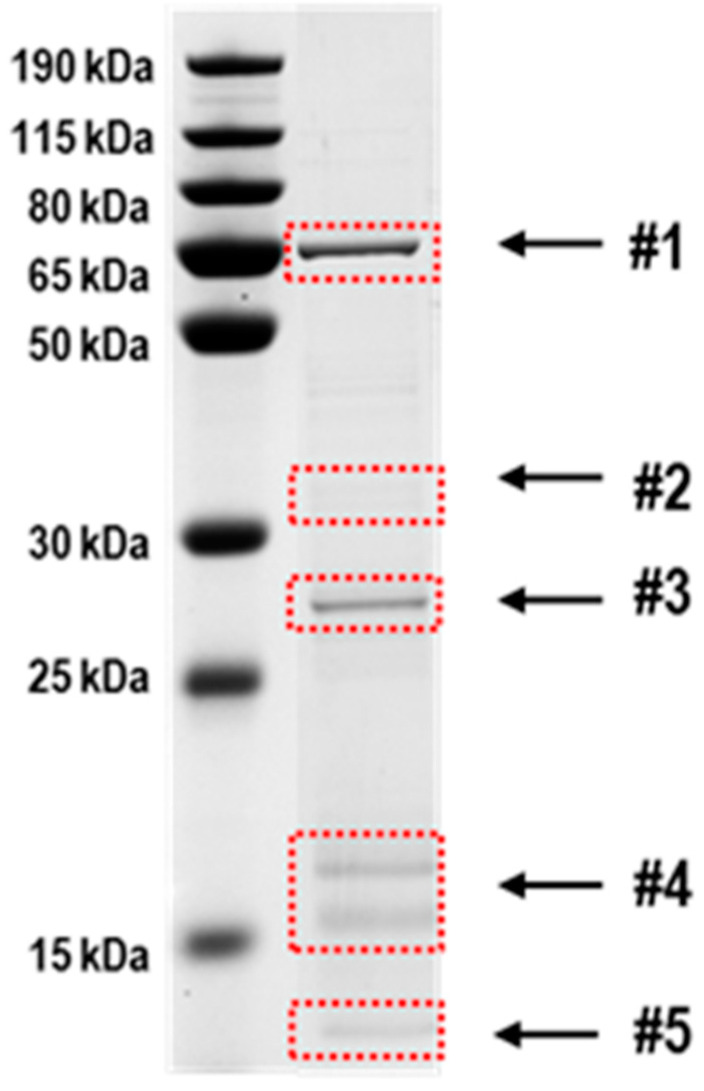
SDS-PAGE profile of the captured proteins by AbxC-affinity chromatography. AbxC was immobilized on CNBr-activated Sepharose 4B resin, incubated with *D. radiodurans* cell lysates, and washed with NaCl-containing buffer. Proteins captured by AbxC were released by 10 mM DTT and then separated on SDS-PAGE. Protein bands were identified after tryptic digestion by mass spectrometry. Table 1 is a complete list of protein bands.

As AbxC was able to form intermolecular disulfide bonds in vitro even in the absence of H_2_O_2_ (Figure 5), we examined whether AbxC could interact with other proteins. FLAG-tagged AbxC was introduced into the chromosome of the Δ*abxC* strain, and whole cell lysates were analyzed by Western blotting. Some protein bands that were not seen in Δ*abxC* were detected by the antibody to FLAG tag in Δ*abxC* expressing FLAG-tagged AbxC, and these bands disappeared after DTT treatment (Figure S5). This suggests that several *D. radiodurans* proteins are physically associated with AbxC, probably through disulfide bond formation. To identify the AbxC-interacting proteins, AbxC was bound to CNBr-activated beads to generate an affinity column, and cell lysates were incubated with AbxC-bound beads. The protein elution with DTT was followed by SDS-PAGE analysis (Figure 6). Bands detected on the gel were excised and subjected to LC-MS/MS analysis. Twenty-five putative AbxC-linked proteins, including AbxC itself, were identified (Table 1).

Overall, the target proteins are involved in diverse metabolic pathways, including glycolysis (DR_1742), NAD^+^ generation (DR_2428), amino acid metabolism (DR_0814, DR_1451, DR_1519), lipid metabolism (DR_1072, DR_1316, DR_A0143), etc. Among them, glucose-6-phosphate isomerase and proline dehydrogenase have been shown to play roles in protecting cells from oxidative stress in *E*. *coli* [43,44]. DR_1298 belongs to the radical-SAM (*S*-adenosyl-l-methionine) superfamily of enzymes, which cleaves SAM to methionine and a potent oxidant 5′-deoxyadenosyl radical [45]. The members of radical-SAM enzymes contain a redox-active [4Fe-4S] cluster ligated by three cysteine residues [45]. It has been hypothesized that BSH plays a role in the Fe–S cluster assembly [19]. One of the prominent family expansions found in *Deinococcus* is the Nudix family of pyrophosphohydrolases, defined as house-cleaning enzymes [46]. These may contribute to radioresistance by removing the deleterious damage products generated by irradiation [46]. Two (DR_0876 and DR_1776) of 23 Nudix hydrolases encoded by *D. radiodurans* were captured by AbxC affinity chromatography (Table 1). Several proteins involved in the oxidative stress response of *D. radiodurans* were also putative target proteins of AbxC. DR_1857 is a homolog of the Cys-based thiol-dependent peroxidase Ohr (organic hydroperoxide resistance protein), which functions as a hydroperoxide reductase, converting organic hydroperoxides to less toxic organic alcohols [10,47]. DR_0615 encodes a proposed novel H_2_O_2_-sensing transcriptional regulator, DrOxyR. In contrast to typical 2-Cys OxyR, which is activated via intramolecular disulfide formation, DrOxyR has a single sensing cysteine (Cys210) residue [6]. DR_1262 encodes a Ro60 ortholog, named DrRsr (Ro sixty-related), which binds misfolded non-coding RNAs and contributes to *D. radiodurans* survival following UV irradiation that can induce ROS generation [48]. Although there is no biochemical information available on DR_1022 encoding a MazG-like nucleotide pyrophosphohydrolase, DR_1022 neighbors the dUTPase DR_2231, which performs housecleaning functions within the framework of oxidative stress response [49]. The formation of an intermolecular disulfide bond probably leads to a conformational change in the protein, and cellular reducing factors (Trx or LMW thiols) cleave the disulfide bond, which regulates protein function [50]. In *D. radiodurans*, IR causes the depletion of BSH, suggesting that BSH is oxidized by IR-induced oxidative stress to maintain cellular redox homeostasis [51]. Since these target proteins are functionally linked to cellular redox status and play roles in antioxidant defense systems, their functions may be affected by redox reactions mediated by AbxC and/or BSH.

### 3.6. AbxC Interacts with DrOxyR

**Figure 7:**
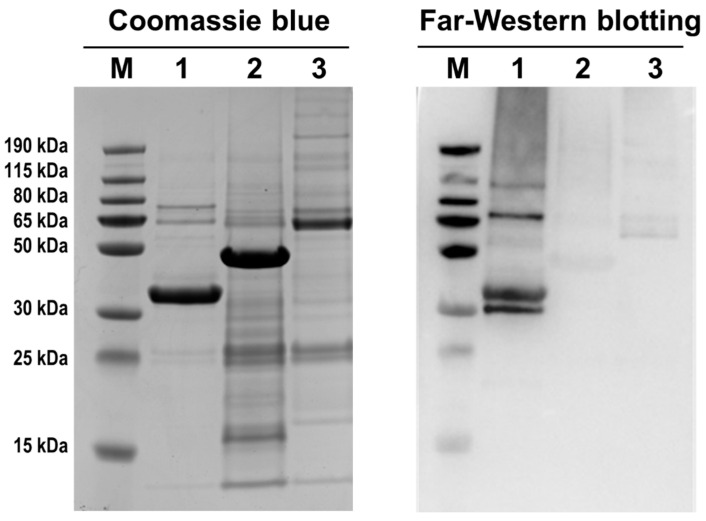
Far-Western blotting of AbxC and its potential target proteins. The recombinant proteins DrOxyR (lane 1), DR_1022 (lane 2), and DrRsr (lane 3) were separated on SDS-PAGE gels and transferred onto PVDF membranes. The purified FLAG-tagged AbxC served as overlay protein. The left panel shows Coomassie blue staining of target proteins, and the right panel shows the corresponding far-Western blot analysis with anti-FLAG antibody.

*D. radiodurans* is extremely resistant to oxidative stress [6]. Among the potential target proteins of AbxC, DrOxyR, DrRsr, and DR_1022, which are known to be associated with oxidative stress response, were chosen for verification to investigate the AbxC role relevant to the H_2_O_2_ resistance of *D*. *radiodurans*. Far-Western blotting was carried out using the recombinant proteins DrOxyR, DrRsr, and DR_1022 to confirm that AbxC interacts with these proteins. Immunoreactive bands were observed in the DrOxyR lane when using FLAG-tagged AbxC as an overlay protein, while no prominent band was observed in other lanes (Figure 7), indicating a direct interaction between DrOxyR and AbxC. DrOxyR proteins do not form intermolecular disulfide linkages with each other, even after H_2_O_2_ treatment, which suggests that DrOxyR can be regulated by modification of the single sensing cysteine residue Cys210 at the post-translational level [6]. Hence, if AbxC interacts with DrOxyR through the formation of a specific disulfide bond between their conserved cysteine residues Cys36 and Cys210, DrOxyR can be turned on and off by AbxC. DrOxyR functions as either a repressor or an activator depending on the redox status of Cys210 [6]. For example, under oxidant stress, oxidized DrOxyR activates *katE1*, whereas under non-stressed conditions, reduced DrOxyR represses *katE1*, in which both oxidized and reduced DrOxyR binds to the *katE1* promoter [8]. AbxCs in their sulfenic acid form may sequester reduced DrOxyR (DrOxyR-SH) in response to H_2_O_2_ treatment, leading to a relative increase in DrOxyR-SOH and a concomitant rise in KatE1. Therefore, the increased sensitivity to H_2_O_2_ of Δ*abxC* might be partly attributable to the dysregulation of DrOxyR (Figure 3A). Further research is warranted.

## 4. Conclusions

**Figure 8:**
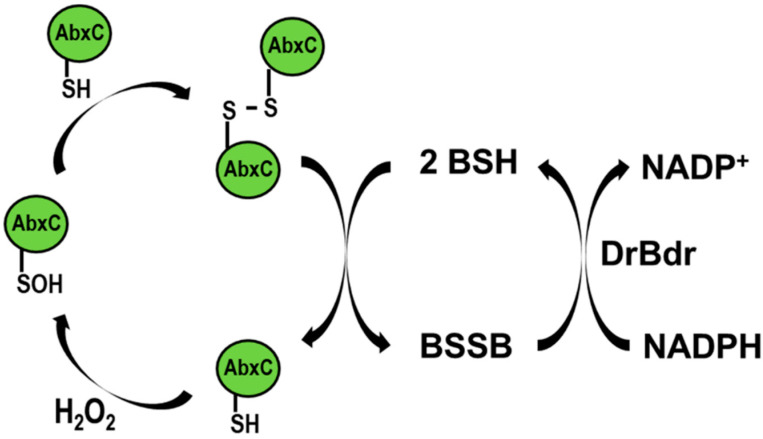
The possible recycling pathway of AbxC. Under H_2_O_2_ stress, oxidation of the AbxC thiol (AbxC-SH) leads to the formation of a sulfenic acid intermediate of AbxC (AbxC-SOH), which can react with another AbxC-SH to form an intermolecular disulfide bond. BSH functions in reduction of the AbxC dimer to release the active AbxC monomer, resulting in BSSB formation. The BSSB reductase DrBdr regenerates BSH on expense of NADPH.

The BSH-dependent redox system consists of NADPH-dependent BSH-specific reductase Bdr, BSH, and BSH-specific oxidoreductase Brx. *D*. *radiodurans* produces BSH and encodes putative BSH-related enzymes. The putative Trx reductase DR_2623 (renamed DrBdr) and Trx family enzyme DR_1832 were suggested to be homologs of Bdr and BrxC, respectively [10]. However, DR_1832 differs from the monothiol BrxC in that it has a TCHKT motif instead of the BrxC TCPIS active motif and a colicin-like immunity domain (PF09204) residing in the C-terminal extension. Therefore, here we named DR_1832 AbxC (atypical BrxC). In this study, we found that the AbxC/BSH/DrBdr/NADPH redox pathway was functional and played a role in the antioxidant defense system against H_2_O_2_, in which DrBdr and AbxC showed BSSB disulfide reductase and BSH-dependent peroxidase activities, respectively (Figure 8). Thiol-oxidation of Cys residues by H_2_O_2_ generates Cys-SOH that can either react with LMW thiols, such as MSH and BSH, or lead to the formation of inter- or intramolecular disulfides in proteins [41]. Cys36, within the TCHKT motif, is the sole cysteine residue of AbxC. H_2_O_2_-treated AbxC proteins appeared as dimers, which were reversible into monomers by BSH, suggesting that disulfide bond formation through Cys36 led to AbxC dimerization. AbxC homologs are present in other bacterial genera, such as *Meiothermus* and *Calidithermus*, which are members of *Deinococcus*–*Thermus* phylum. The sequence identity between the *D. radiodurans* AbxC and the other AbxCs ranges from 51% (AbxC of *Meiothermus hypogaeus*) to 57% (AbxC of *Calidithermus timidus*) (data not shown), suggesting that the BSH-dependent peroxidase AbxC may contribute to the resistance to environmental stresses in these species. In addition, AbxC interacted with proteins involved in cellular metabolism and the *D. radiodurans* peroxide-sensing transcriptional regulator DrOxyR. This implies that AbxC participates in the H_2_O_2_-signaling pathway. Recently, it was found that *B. subtilis* BrxC (previously named YtxJ) can catalyze the de-bacillithiolation of several proteins [23]. The possibility of the dual function of AbxC as peroxidase in response to H_2_O_2_ and as Brx in the de-bacillithiolation pathway cannot be ruled out because AbxC is the only homolog of Brx found in *D*. *radiodurans*. However, it has been suggested that other oxidoreductases, including the essential Trx system, might contribute to de-bacillithiolation in *B. subtilis* [20]. Therefore, there is a need to identify *S*-bacillithiolated proteins in *D*. *radiodurans* and to investigate the role of AbxC in the removal of BSH from the proteins.

## Figures and Tables

**Table 1:** Potential AbxC interacting proteins.

Fraction	Locus tag	Protein	Size	Count *	No. of Cys
#1	DR_1262	60 kDa SS-A/Ro ribonucleoprotein	57 kDa	5	2
	DR_1298	radical SAM superfamily	58 kDa	6	5
	DR_1316	propionyl-CoA carboxylase	57 kDa	10	4
	DR_1742	glucose-6-phosphate isomerase	60 kDa	3	2
#2	DR_0615	transcriptional regulator OxyR	35 kDa	3	1
	DR_0814	proline dehydrogenase	35 kDa	6	2
	DR_1072	acetyl-CoA acetyltransferase	41 kDa	7	4
	DR_1022	NTP pyrophosphatase MazG	37 kDa	8	3
	DR_1519	ketol-acid reductoisomerase	37 kDa	13	3
	DR_1890	oxidoreductase	35 kDa	3	4
	DR_2428	nicotinamide-nucleotide adenylyltransferase	38 kDa	3	1
	DR_A0143	3-hydroxyacyl-CoA dehydrogenase	38 kDa	3	1
#3	DR_0114	enoyl-CoA hydratase, putative	27 kDa	7	2
	DR_1451	*S*-adenosylhomocysteine nuclosidase	25 kDa	4	2
	DR_1832	putative homolog of BrxC (YtxJ)	24 kDa	13	1
#4	DR_0763	predicted acetyltransferase	18 kDa	3	1
	DR_0876	Nudix family	18 kDa	3	2
	DR_1245	putative sensory transduction regulator	19 kDa	6	1
	DR_1418	signal transduction response regulator	15 kDa	5	2
	DR_1654	hypothetical protein	19 kDa	5	3
	DR_1776	Nudix family	19 kDa	4	3
	DR_1857	organic hydroperoxide reductase	15 kDa	3	2
	DR_2298	acyl-CoA thioester hydrolase	19 kDa	4	2
	DR_2481	hypothetical protein	17 kDa	5	1
#5	DR_2580	ribosomal silencing factor RsfS	13 kDa	6	1

* The number of spectra matched to peptides from a protein.

## Data Availability

Data is contained within the article and supplementary material.

## Supplementary Materials

The following are available online at https://www.mdpi.com/article/10.3390/antiox10071148/s1, Table S1: Primers used in this study, Figure S1: Multiple alignment of amino acid sequences of *D. radiodurans* Bdr (DR_2623) and its homologs, Figure S2: Multiple alignment of amino acid sequences of deinococcal AbxC homologs, Figure S3: Growth curve of *bshA* mutant, Figure S4: NADPH consumption by the AbxC/BSH/DrBdr pathway in response to H_2_O_2_, and Figure S5: Western blotting of *D. radiodurans* cells with FLAG-tagged AbxC.
