# Supplementary material for: Atypical Bacilliredoxin AbxC Plays a Role in Responding to Oxidative Stress in Radiation-Resistant Bacterium Deinococcus radiodurans
Source: Antioxidants (Basel). 2021 Jul 20;10(7):1148. doi: 10.3390/antiox10071148 (PMC8301015; doi:10.3390/antiox10071148)
Supplement: Supplementary file 1 [file antioxidants-10-01148-s001.zip › antioxidants-1278152-supplementary.pdf]

Supplementary Table S1. Primers used in this study

Primer	Sequences (5′→3′)*	RE
Mutant construction		
1555-UP-F	attctcgagcagggtgccgaccatt	XhoI
1555-Up-R	gtatgatatcggtggcgaccacgccgg	EcoRV
1555-Dw-F	aaggatcccgaagtcgtgcagcacg	BamHI
1555-Dw-R	tatctgcagcacgccgcccacctg	PstI
1832-Up-F	attctcgagtggctgacccgcttaca	XhoI
1832-Up-R	gtatgatatccttcagaaactggtccacctcg	EcoRV
1832-Dw-F	aaggatccgacgcctaccacggcg	BamHI
1832-Dw-R	tatctgcagcggcaact gccggg	PstI
1832-Flag-F	cgggtaagctgtcggcatggactacaaagaccatgacggtgat	
1832-Flag-R	tggtctttgtagtccatgccgacagcttacccgaatgc	
Plasmid construction		
1832-F	taagggcccatgacccaaggccaagaccaa	ApaI
1832-R	gtatgatatctcagccgagctggtcgagca	EcoRV
C36S-F	atggtcttgtggctggtgccggccttg	
C36S-R	caaggccggcaccagccacaagaccat	
Protein purification		
1832-His-F	gatgcggccgcaacccaaggccaagaccaagtgc	NotI
1832-His-R	gattctcgaggccgagctggtcgagcacg	XhoI
2623-His-F	attcatatgagtctttatgacgtggcaa tcgtcggagcc	NdeI
2623-His-R	attctcgagggccgggcgcagttcgccg	XhoI
0615-His-F	gattcatatggaactgcgacacctgcg	NdeI
0615-His-R	gattctcgagcctgggcggctcggtgggca	XhoI
1022-His-F	gattcatatgaccaccccaaccttctcg	NdeI
1022-His-R	tatgtcgaccttcgggcacttcggtgacga	SalI
1262-His-F	gattcatatgaagaacttgctccgtgccatc	NdeI
1262-His-R	gattctcgagaacctcgccccgcgcaaaag	XhoI

* Restriction enzyme (RE) recognition sites are underlined in the sequences.

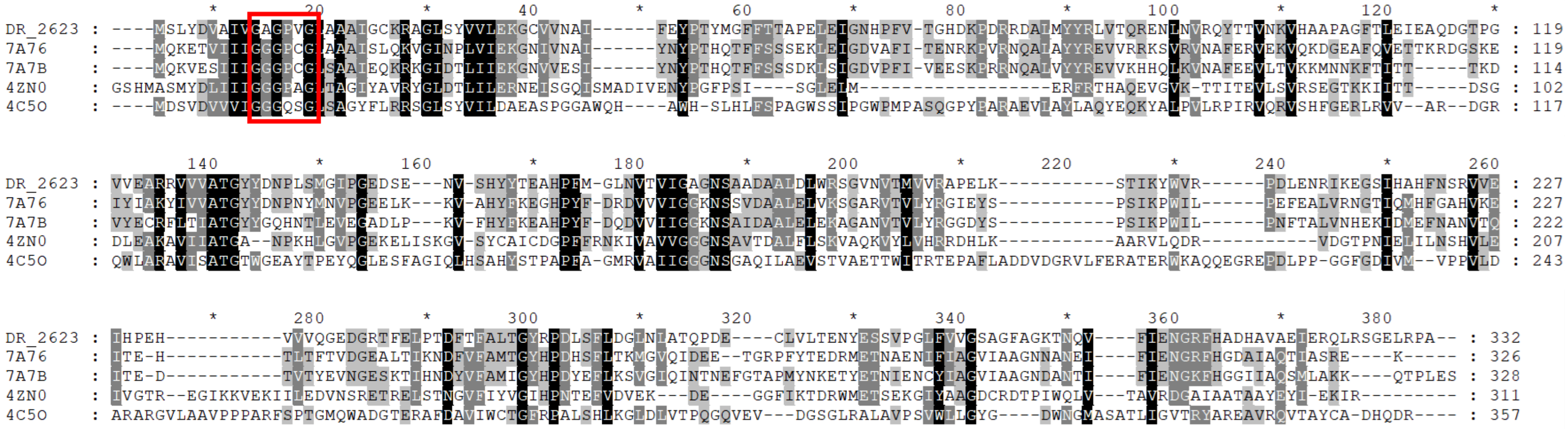

Supplementary Figure S1. Multiple alignment of amino acid sequences of *D. radiodurans* Bdr (DR_2623) and its homologs. The conserved GXGXXG motifs are indicated by red line. The program Genedoc (www.psc.edu/ biomed/genedoc) was used to visualize the alignment in quantify mode, which highlights residues most-frequently found in each column of the alignment. Black letters on light gray shading represent 60% identity. White letters on gray and black shading represent 80% and 100% identities, respectively. Protein sequences obtained from the Protein Data Bank (PDB) are distinguished by the PDBid, 7A76; *Bacillus cereus*, 7A7B; *Staphylococcus aureus*, 4ZN0; *Metanosarcina maezi*, 4C5O; *Stenotrophomonas maltophilia*.

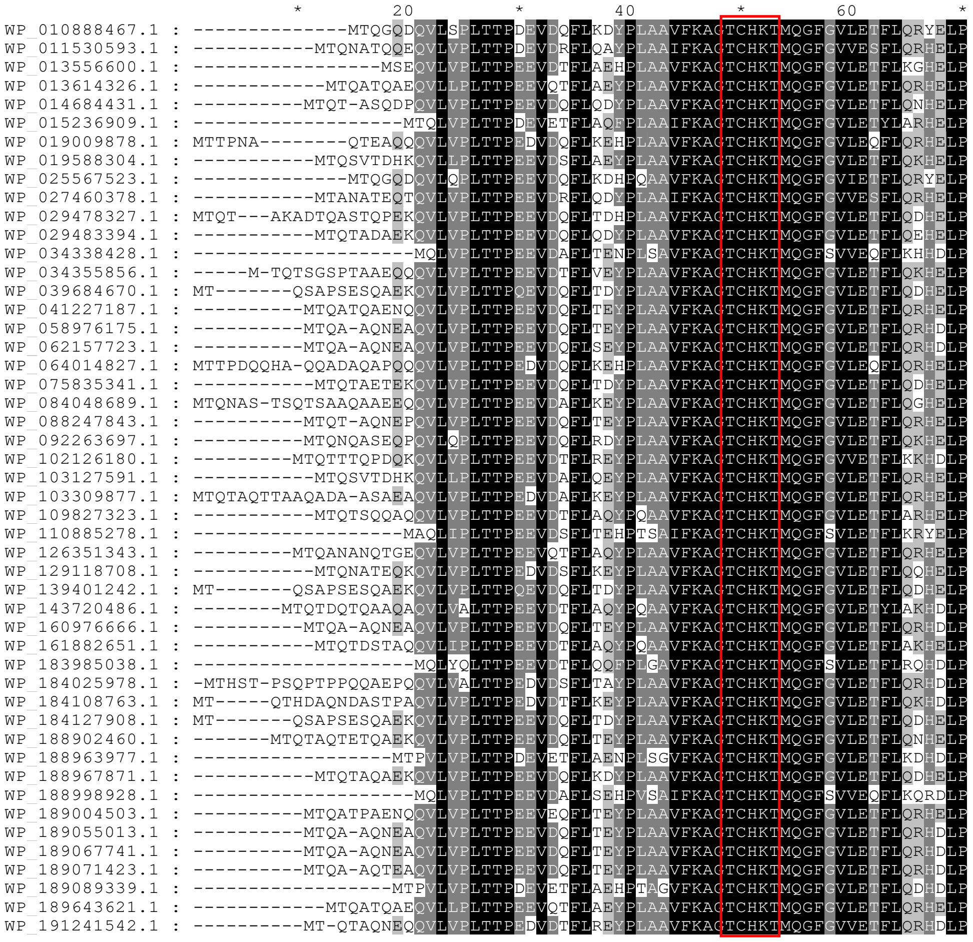

Supplementary Figure S2. Multiple alignment of amino acid sequences of deinococcal AbxC homologs. The TCHKT and its neighboring residues of 49 AbxC homologs are aligned, and the conserved TCHKT motifs are indicated by red line. Protein sequences obtained from the NCBI database are distinguished by the WP protein accession number. Black letters on light gray shading represent 60% identity. White letters on gray and black shading represent 80% and 100% identities, respectively.

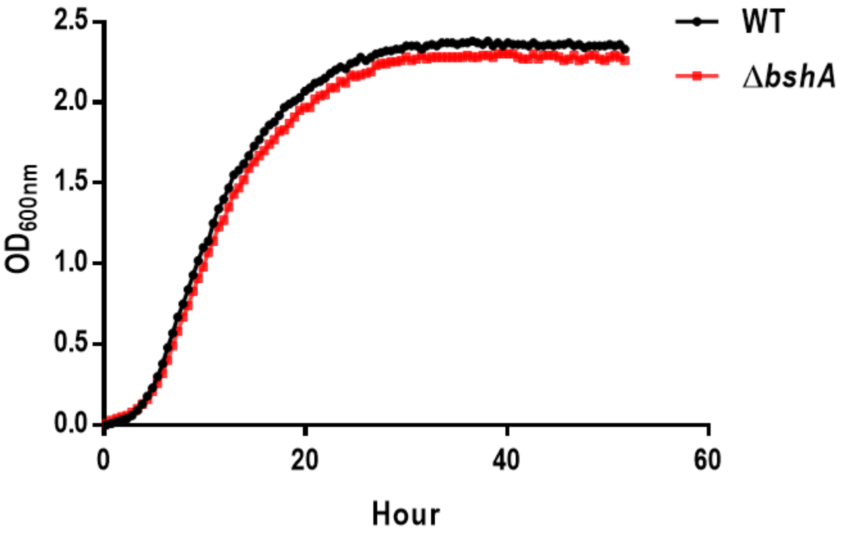

Supplementary Figure S3. Growth curve of *bshA* mutant. The wild-type (WT) and *bshA* mutant (Δ*bshA*) cells grown to the stationary phase in the TGY medium were inoculated at 1:100 into a fresh TGY medium, samples were taken regularly during the incubation from cultures of cells. The optical densities were monitored at 600 nm. The results are representative of two independent experiments.

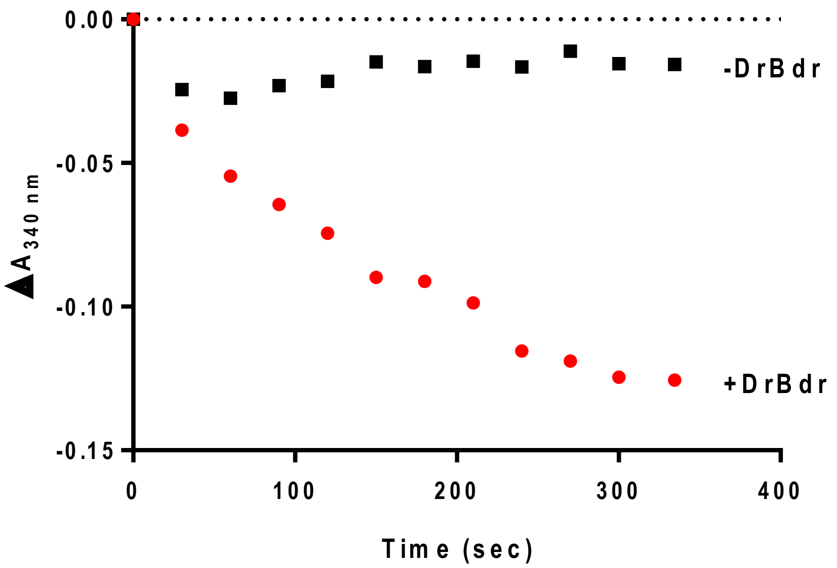

Supplementary Figure S4. NADPH consumption by the AbxC/BSH/DrBdr pathway in response to H_2O_2. The initial reaction mixtures contained 500 μM BSH, 500 μM NADPH, and 10 μM of AbxC. The NADPH consumption was monitored at 340 nm in the absence of DrBdr (– DrBdr) or in the presence of 0.5 μM of DrBdr (+ DrBdr) immediately after addition of 10 mM H_2O_2. The experiments were repeated twice and representative data are shown.

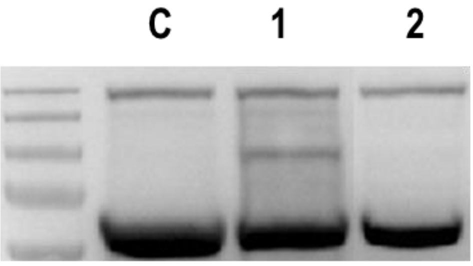

Supplementary Figure S5. Western blotting of *D. radiodurans* cells with FLAG-tagged AbxC. Soluble proteins were extracted from Δ*abxC* (C, control), Δ*abxC* expressing FLAG-tagged AbxC (lane 1), and Δ*abxC* expressing FLAG-tagged AbxC following 10 mM DTT treatment (lane 2). Five microgram protein samples were separated on non-reducing SDS-PAGE and blotted onto nitrocellulose membrane. AbxC-interacting proteins were detected using the anti-FLAG antibodies.
